# Association of Personal Characteristics and Effectiveness of Immunotherapy in Late-Stage Non-Small Cell Lung Cancer: A Systematic Review

**DOI:** 10.1093/jncics/pkac015

**Published:** 2022-02-17

**Authors:** Krishna Patel, Naomi Alpert, Stephanie Tuminello, Emanuela Taioli

**Affiliations:** 1 Institute for Translational Epidemiology and Department of Population Health Science and Policy, Icahn School of Medicine at Mount Sinai, New York, NY, USA; 2 Division of Epidemiology, New York University Grossman School of Medicine, New York, NY, USA; 3 Institute for Translational Epidemiology and Department of Population Health Science and Policy, Icahn School of Medicine at Mount Sinai, Tisch Cancer Institute, New York, NY, USA

## Abstract

**Background:**

Although immunotherapy can increase survival in non-small cell lung cancer (NSCLC), response rates are low. It is unclear which characteristics contribute to variability in immunotherapy efficacy and survival. Research is needed to identify reasons for heterogeneity in response rates to better tailor treatments.

**Methods:**

Web of Science, Ovid EMBASE, and MEDLINE were queried from 2013 to January 2021, and all studies reporting overall or progression-free survival for patients treated with immunotherapy for NSCLC of at least stage IIIB were screened.

**Results:**

Included were 18 randomized controlled trials (RCTs; 6534 immunotherapy RCTs; 11 192 nonimmunotherapy RCTs) and 16 observational studies (n = 9073 immunotherapy patients). Among RCTs, there was improved survival with the addition of immunotherapy in patients aged younger than 65 years in 10 of 17 studies; smokers in 8 of 15 studies; and males in 10 of 17 studies and 6 of 17 females. Only 5 studies reported outcomes by race. Among observational studies, younger patients (aged younger than 60, younger than 65, or younger than 70 years in most studies) had better survival than older patients (aged 60 years and older, 65 years and older, or 70 years and older) in 4 of 13 studies, ever-smokers in 7 of 13, and females in 2 of 14. Three studies reported race with mixed results.

**Conclusion:**

Although evidence is mixed, younger patients, smokers, and males may derive more benefit from immunotherapy. Evidence on racial differences is limited. Physicians should be mindful of personal characteristics when formulating treatment plans. Further research is needed to understand underlying mechanisms and to identify the best immunotherapy candidates and alternative treatments for those unlikely to benefit.

Immunotherapy is now the standard of care for many of the approximately 200 000 non-small cell lung cancer (NSCLC) patients diagnosed annually in the United States (1). For some late-stage, unresectable cancers, immunotherapies can be less toxic and more targeted than chemotherapy. Multiple approaches combining immune checkpoint inhibitors with chemotherapy and radiotherapy continue to be tested (2). Compared with other cancers, NSCLC is relatively amenable to immunotherapy, but many patients still do not benefit. Reported response rates for pembrolizumab and nivolumab for patients with Programmed death—ligand 1 (PD-L1) of more than 50% are 41% and 19%, respectively, and as low as 15% for atezolizumab (3). Research is ongoing to identify personal characteristics, biomarkers, and clinical or histological features that may explain heterogeneity in immunotherapy response and predict which patients may benefit most.

However, much remains poorly understood about how various prognostic factors contribute to differences in efficacy and disparities in survival among those receiving immunotherapy, especially in the presence of clinical characteristics that can act as confounders. There is substantial heterogeneity of clinical characteristics, tumor mutation expression, and line of therapy between existing studies, which heightens the need for synthesis across experimental and observational studies. Patients enrolled in clinical trials tend to be White, younger, healthier, and more tolerant of treatment-related toxicities (4). Women have long been underrepresented in clinical trials (4), as have minority groups, and there has not been a systematic attempt to aggregate evidence on metastatic NSCLC with respect to race (5). Concurrently, increased use of immunotherapy globally has enabled publication of multiple observational studies with more diverse patients and more representative, real-world outcomes. This systematic review aims to synthesize evidence across experimental and observational studies to assess the effects of age, smoking status, sex, and race on survival among those receiving immunotherapy.

## Methods

### Search Strategy

Ovid EMBASE, MEDLINE (including PubMed), and Web of Science were queried using MeSH and free-text terms identified using the Population-Intervention-Comparator-Outcomes—Timing—Setting framework (6). Search logic was [(Immuno* OR CHECK* OR PD-1 OR PD-L1 OR *mab OR cyramza)] AND TI=(NSCLC OR “non-small cell lung cancer” OR “non-small cell lung cancer”) AND TS=(“sex” OR “gender” OR “smok*” OR “rac*” OR “ethnic*” OR “age” OR “Comorb*” OR “soci*” OR “socioec*”), as a topic (title, abstract, keyword search) search in Web of Science, and a title-only search in Ovid. Food and Drug Administration approval was granted for immunotherapy in NSCLC in 2015; studies from 2013 to January 2021 were included to provide a 2-year buffer. Bibliographies of all review articles returned by the searches were hand searched and evaluated for inclusion.

### Inclusion and Exclusion Criteria and Selection of Studies

All clinical trials, observational studies, and secondary analyses studying patients diagnosed with late-stage NSCLC, regardless of histology, that reported hazard ratios (HRs) for either overall or progression-free survival were eligible for inclusion.

Studies were also eligible for inclusion if they reported hazard ratios for overall survival (OS) or progression-free survival (PFS) (or data that allowed for calculation of these values) and had immunotherapy and nonimmunotherapy treatment arms. Ongoing trials as of January 31, 2021, trial protocols, case reports, and data on patient samples were excluded.

### Data Collection and Extraction

Search results were exported into EndNote X9 (Clarivate, Endnote X9) (7), and all duplicates were removed. Titles and abstracts were assessed for eligibility, and full-text articles were referenced when no exclusion criteria were met during this initial screen.

For experimental studies, the following data were extracted: prognostic factor of interest, hazard ratio for all patients, hazard ratio stratified by prognostic factor subgroups, histology, line of treatment, stage, and immunotherapy and control treatments. For immunotherapy groups, the number of patients, percent male, and median age were extracted. For observational studies, extracted data included the following: prognostic factor of interest, immunotherapy used, lines of treatment, survival outcome (OS or PFS), univariate hazard ratio, multivariate hazard ratio, covariate adjustments, stage, histology, and median age.

Data extraction forms for age, smoking status, sex, and race differed slightly based on the way each prognostic factor was categorized. Data about race were not extracted if more than 90% of study participants were of one ethnicity, and hazard ratios for minority subgroups were not reported. Data on race extracted as “Other” refers to studies where “Other” was used by the authors as a category without further breakdown. Quality review of all included experimental and observational studies was completed by 2 reviewers who scored the studies independently, using National Institutes of Health–developed tools (8,9). All discrepancies were resolved between the 2 reviewers, and a third, independent reviewer confirmed the final judgements (Supplementary Tables 1 and 2, available online).

## Results

### Search Results

The search and eligibility screening process is illustrated in Supplementary Figure 1 (available online). After removing duplicates, 1124 titles and abstracts were screened. The full texts of 65 studies were assessed along all inclusion and exclusion criteria; 18 experimental studies (all randomized controlled trials [RCTs]) and 16 observational studies met all inclusion criteria (Tables 1 and 2).

**Table 1.** Summary of included experimental studies (n = 18)

Clinical trial	Author, year	Prognostic factors	Histology	Line of therapy	Stage	Immunotherapy	Control	No. immunotherapy/total	Male, %	Female, %	NIH rating (out of 14)
KEYNOTE-010	Herbst et al., 2016 (10)	Age, sex	NSCLC (22% squamous)	Second	Advanced	Pembrolizumab (10 mg/kg or 2 mg/kg—pooled)	Docetaxel (75 mg/m^2^)	690/1033	62	38	12
KEYNOTE-024^a^	Reck et al., 2016 (11)	Age, smoking, sex	NSCLC (19% squamous)	First	IV	Pembrolizumab (200 mg)	Platinum	154/305	60	40	13
KEYNOTE-042	Mok et al., 2019 (12)	Age, smoking, sex	NSCLC (38% squamous)	First	Locally advanced or metastatic	Pembrolizumab (200 mg)	Platinum	637/1274	71	29	12
KEYNOTE-189	Gandhi et al., 2018 (13)	Age, smoking, sex	Nonsquamous	First	Metastatic	pembrolizumab (200 mg) + pemetrexed + platinum	Platinum	410/616	62	38	14
KEYNOTE-407	Paz-Ares et al., 2018 (14)	Age, sex	Squamous	First	IV	Pembrolizumab (200 mg) + paclitaxel/nab-paclitaxel + carboplatin	Platinum	278/559	79	21	14
IMpower130	West et al., 2019 (15)	Age, smoking, sex	Nonsquamous	First	IV	Atezolizumab (1200 mg) + carboplatin + nab-paclitaxel	Platinum	451/723	59	41	10
IMpower131	Jotte et al., 2020 (16)	Age, smoking, sex, race	Squamous	First	IV	Atezolizumab (1200 mg) + carboplatin + paclitaxel/nab-paclitaxel	Carboplatin + paclitaxel/nab-paclitaxel	343/683	82	18	10
IMpower132	Nishio et al., 2020 (17)	Age, smoking, sex, race	Nonsquamous	First	IV	Atezolizumab (1200 mg) + cisplatin/carboplatin + pemetrexed	Carboplatin/cisplatin + pemetrexed	292/578	66	34	12
IMpower150^a^	Socinski et al., 2018 (18)	Age, smoking, sex	Nonsquamous	First	IV or recurrent	Atezolizumab + carboplatin + paclitaxel + bevacizumab	Carboplatin + paclitaxel + bevacizumab	356/692	60	40	11
CheckMate 017	Brahmer et al., 2015 (19)	Age, smoking, sex	Squamous	Second	IIIB: 21%; IV: 79%	Nivolumab (3 mg/kg)	Docetaxel (75 mg/m^2^)	135/272	82	18	12
CheckMate 026	Carbone et al., 2017 (20)	Age, smoking, sex	NSCLC	First	IV or recurrent	Nivolumab (3 mg/kg)	Platinum	271/541	68	32	10
CheckMate 057	Borghaei et al., 2015 (21)	Age, smoking, sex	Nonsquamous	Second	IIIB: 8%; IV: 92%	Nivolumab (3 mg/kg)	Docetaxel (75 mg/m^2^)	292/582	52	48	11
CheckMate 227	Hellmann et al., 2019 (22)	Age, smoking, sex	NSCLC	First	IV/Recurrent	Nivolumab (3 mg/kg) + ipilimumab (1 mg/kg)	Platinum doublet chemo-therapy	396/793	64	36	12
OAK	Rittmeyer et al., 2017 (23)	Age, smoking, sex	NSCLC	Second or third	IIIB-IV	Atezolizumab (1200 mg)	Docetaxel (75 mg/m^2^)	425/850	61	39	11
JAVELIN Lung 200	Barlesi et al., 2018 (24)	Age, smoking, sex, race	NSCLC (30.5% squamous)	Second	IIIB-IV/Recurrent NSCLC	Avelumab (10 mg/kg)	Docetaxel (75 mg/m^2^)	396/792	69	31	11
CA184-104	Govindan et al., 2017 (25)	Age, sex, race	NSCLC	First	IV/Recurrent	Ipilimumab + carboplatin + paclitaxel	Placebo + carboplatin + paclitaxel	388/749	84	16	13
PACIFIC	Antonia et al., 2018 (26)	Age, smoking, sex, race	NSCLC	Second and further	Stage III unresectable	Durvalumab + chemo-radiotherapy	Placebo + chemo-radiotherapy	476/713	70	30	14
POPLAR	Fehrenbacher et al., 2016 (27)	Smoking	NSCLC (34% squamous)	Second-third	All comers	Atezolizumab (1200 mg)	Docetaxel (75 mg/m^2^)	144/287	65	35	12

a Studies reporting outcomes as progression-free survival. The remaining studies reported overall survival. NIH = National Institutes Health; NSCLC = non-small cell lung cancer.

**Table 2.** Summary of included observational studies (n = 16)^a^

Study	Study population	Prognostic factors	Histology	Line of therapy	Stage	Immunotherapy	No. of patients	Male, %	Female, %	NIH rating
Chen et al., 2020 (28)^a^	Peking Union Medical College Hospital (China)	Age, smoking, sex	Nonsquamous: 59.8%; squamous: 40.2%	Second (74%); Third (26%)	IIIB: 22.68%; IV: 77.32%	Pembrolizumab (35/97) or nivolumab (62/97)	97	67	33	10/14
Smit et al., 2020 (29)	Netherlands NVALT Registry	Age, smoking	ADC: 66.1%; SCC: 26.6%; NOS: 6.3%	All	IV: 100%	Nivolumab and pembrolizumab for second line; durvalumab, atezolizumab	2302	57	43	10/14
Lichtenstein et al., 2019 (30)	Massachusetts General	Age, sex	ADC: 65.3%; SCC: 25.7%; LCC: 1.6%; other: 7.3%	All	I: 10.6%; II: 7.8%; III: 16.3%; IV: 62.9%; Unknown: 2.5%	PD-1/PD-L1 inhibitors	141	54	46	10/14
Yang et al., 2020 (31)^a^	Shandong Cancer Hospital	Age, smoking, sex	ADC: 46.5%; SCC: 53.5%	Third-anlotinib (RTK-inhibitor targeting VEGF2/3) + immunotherapy; ECOG PS 0-2	III: 43.5%; IV: 56.5%	Anlotinib + immunotherapy combination (pembrolizumab [41], sintilimab [32], 15 nivolumab [15], tislelizumab [13])	101	58	42	10/14
Huang et al., 2020 (32)	Harbin Medical University Cancer Hospital (China)	Age, smoking, sex	ADC: 73.2%; SCC: 26.8%	All (first [28%]; second and later [72%]), patients receiving at least 1 cycle ICI and wild-type EGFR, ALK, ROS	IIIB: 9.8%; IV: 90.2%	Anti-PD-1 (nivolumab and pembrolizumab); anti-PD-L1 (Atezolizumab); CTLA4 antibody (Ipilimumab)	61	62	38	10/14
Nazha et al., 2020 (5)	Winship Cancer Institute, Emory University	Sex, race	ADC: 65.2%; SCC: 17.8%; large cell lung cancer: 2%; NOS: 6.7%; SCLC: 7.1%	All (first [19.9%], second [62.9%], third [12.9%], fourth [4.3%])	III: 19.8%; IV: 67.8%; nonadvanced: 13.4%	Single agent (nivolumab [49%], pembrolizumab [25.3%], atezolizumab [21.4%], nivolumab + ipilimumab [4.3%])	257	49	51	10/14
Prelaj et al., 2019 (33)^a^	National Cancer Institute of Milan	Age, smoking, sex	ADC: 73%; SCC 24%; other: 3%	Second (61%) or further (39%)	IIIB-C: 3%; IV: 97%	Anti-PD 1/anti PD-L1 (single agent)	193	62	38	10/14
Elkrief et al., 2020 (34)	Dijon Cancer Center (n = 177); University of Montreal University Hospital (n = 106); Quebec Heart and Lung Institute (n = 98)	Age, smoking, sex	Nonsquamous: 73%; SCC: 22%, other: 5%	First (12%); second or further (88%)	III: 7%; IV: 93%	Nivolumab (67%), pembrolizumab (28%), other (5%)	381	57	43	10/14
Kano et al., 2020 (35)	Okayama Lung Cancer Database (Japan)	Smoking, sex	ADC: 63.9%; SCC: 28.3%; other: 7.7%	First (17%); second (35%); third or further (48%)	IIIB-IV: 71.7%; recurrence: 28.3%	Anti-PD-1/Anti-PD-L1 monotherapy (nivolumab [69.8%]; pembrolizumab [29.6]; atezolizumab 0.6%)	527	79	21	10/14
Anouti et al., 2020 (36)	Hoosier Cancer Research Network (LUN 14-19 trial)	Age, smoking, sex	Nonsquamous: 55%; squamous: 45%	Previous chemoradiation	IIIA: 60%; IIIB: 40%	Pembrolizumab	92	64	36	10/14
Adachi et al., 2019 (37)^a^	Kinki-Chuo Chest Medical Center (Japan)	Age, smoking, sex	ADC: 62.5%; SCC: 27.4%; other: 10.1%	All	NR	Nivolumab	296	70	30	10/14
Ahn et al., 2019 (38)	Yonsei Cancer Center, Korea	Age, smoking, sex	ADC: 67.7%; SCC: 30.3%; other: 2%	All (first [10.3%], second: [39.4%], third or further [50.3%])	100% advanced NSCLC	Nivolumab, pembrolizumab	155	73	27	10/14
Lin et al., 2018 (39)	National Taiwan University Hospital	Age, smoking, sex	ADC: 64.9%; SCC: 18.9%; other: 16.2%	All (69% Third- or further-)	IIIB: 2.7%, IV: 97.3%	Nivolumab or pembrolizumab	74	58	42	10/14
Ng et al., 2018 (40)^a^	University of Colorado Hospital and Shanghai Pulmonary Hospital, Tongji University	Age, smoking, sex, race	NR	All	100% locally advanced or metastatic NSCLC	Anti-PD-1/PD-L1 monotherapy—nivolumab, pembrolizumab, atezolizumab	91	NR	NR	10/14
Foster et al., 2019 (41)	US National Cancer Database	Age, sex, race	ADC: 79.1%; non-ADC: 20.9%	First commission on cancer-accredited programs	IV: 100%	Unspecified (most likely pembrolizumab)	5807	52	48	10/14
Song et al., 2020 (42)^a^	Peking Union Medical College	Smoking	ADC: 42.86%; SCC: 57.14%	All (first [32]; second [22]; third and further [9])	IIIB: 6.35%; IIIC: 9.52%; IVA: 61.9%; IVB: 22.22%	Pembrolizumab (42), nivolumab (4), sintilizumab (17)	63	84	16	10/14

a Studies reporting outcomes as progression-free survival. The remaining studies reported overall survival. ADC = adenocarcinoma; ALK = Anaplastic lymphoma kinase; anti PD-L1 = anti Programmed death-ligand 1; CTLA4 = Cytotoxic T-lymphocyte-associated antigen 4; ECOG PS = Eastern Cooperative Oncology Group Performance Status; EGFR = epidermal growth factor receptor; ICI = immune checkpoint inhibitor; LCC = large cell carcinoma; NIH = National Institutes of Health; NOS = not otherwise specified; NVALT = Nederlandse Vereniging voor Artsen Longziekten en Tuberculose; ROS = Reactive oxygen species; RTK = Receptor tyrosine kinase; SCC = squamous cell carcinoma; VEGF = Vascular endothelial growth factor.

### Quality Assessment

All 18 included experimental studies fulfilled at least 10 of 14 checklist items; all were randomized, had groups comparable across all covariates at baseline, and used reliable outcome measures (9). Although many of the included studies were open-label and unblinded, the focus on hard survival outcomes reduces the potential for bias in outcome measurements. Multiple studies did not report details about allocation concealment (Supplementary Table 1, available online) (15,16,18,23,24). All 18 experimental studies specified a priori any patient subgroup analyses for survival outcomes.

All 16 observational studies met 10 of 14 checklist items (9). Areas of potential bias included retrospective analyses, lack of sample size justifications, and sparse reporting around whether hypotheses were specified a priori (Supplementary Table 2, available online).

### Narrative Synthesis of Experimental Studies

The 18 included experimental studies had immunotherapy treatment regimens of pembrolizumab (n = 5), atezolizumab (n = 6), nivolumab (n = 4), durvalumab (n = 1), avelumab (n = 1), and ipilimumab (n = 1) (Table 1). Two studies reported hazard ratios based on PFS, and 16 studies reported hazard ratios for OS. Nine studies tested chemotherapy–immunotherapy combinations, and the other 9 compared single-agent immunotherapy to chemotherapy. All control groups consisted of 1 or more chemotherapy drugs including docetaxel, paclitaxel, and various platinum chemotherapies. Patients were enrolled in first (n = 11), second (n = 4), or second and/or third (n = 3) lines of treatment (23,26,27). Of the 18 included experimental studies, 13 reported statistically significant improvements in OS or PFS with immunotherapy across all patients (Table 3).

**Table 3.** Association between survival and immunotherapy, stratified by age in experimental studies (n = 17)

Clinical Trial	Study	Immunotherapy vs no immunotherapy by age				
		HR (95% CI)				
		All patients	Younger than 65 years	65 years and older	65-75 years	Older than 75 years
KEYNOTE-024	Reck et al., 2016 (11)^a^	0.50 (0.37 to 0.68)	0.61 (0.40 to 0.92)	0.45 (0.29 to 0.70)	N/A	N/A
KEYNOTE-042	Mok et al., 2019 (12)	0.81 (0.71 to 0.93)	0.81 (0.67 to 0.98)	0.82 (0.66 to 1.02)	N/A	N/A
KEYNOTE-189	Gandhi et al., 2018 (13)	0.49 (0.38 to 0.64)	0.43 (0.31 to 0.61)	0.64 (0.43 to 0.95)	N/A	N/A
KEYNOTE-407	Paz-Ares et al., 2018 (14)	0.64 (0.49 to 0.85)	0.52 (0.34 to 0.80)	0.74 (0.51 to 1.07)	N/A	N/A
IMpower130	West et al., 2019 (15)	0.79 (0.64 to 0.98)	0.79 (0.58 to 1.08)	0.78 (0.58 to 1.05)	N/A	N/A
IMpower131	Jotte et al., 2020 (16)	0.88 (0.73 to 1.05)	0.89 (0.68 to 1.15)	N/A	0.84 (0.63 to 1.13)	0.74 (0.45 to 1.23)
IMpower132	Nishio et al., 2020 (17)	0.86 (0.71 to 1.06)	0.88 (0.67 to 1.16)	0.84 (0.63 to 1.13)	N/A	N/A
IMpower150	Socinski et al., 2018 (19)^a^	0.62 (0.52 to 0.74)	0.65	N/A	0.52	0.78
CheckMate 017	Brahmer et al., 2015 (19)	0.59 (0.44 to 0.79)	0.52 (0.35 to 0.75)	N/A	0.56 (0.34 to 0.91)	1.85 (0.76 to 4.51)
CheckMate 026	Carbone et al., 2017 (20)	1.08 (0.87 to 1.34)	1.13 (0.83 to 1.54)	1.04 (0.77 to 1.41)	N/A	N/A
CheckMate 057	Borghaei et al., 2015 (21)	0.75 (0.62 to 0.91)	0.81 (0.62 to 1.04)	N/A	0.63 (0.45 to 0.89)	0.90 (0.43 to 1.87)
KEYNOTE-010	Herbst et al., 2016 (10)	0.67 (0.56 to 0.80)	0.63 (0.50 to 0.79)	0.76 (0.57 to 1.02)	N/A	N/A
OAK	Rittmeyer et al., 2017 (23)	0.73 (0.62 to 0.87)	0.80 (0.64 to 1.00)	0.66 (0.52 to 0.83)	N/A	N/A
JAVELIN Lung 200	Barlesi et al., 2018 (24)	0.90 (0.73 to 1.12)	0.84 (0.63 to 1.13)	0.98 (0.71 to 1.34)	N/A	1.16 (0.54 to 2.47)
CA184-104	Govindan et al., 2017 (25)	0.91 (0.77 to 1.07)	0.82 (0.64 to 1.04)	N/A	1.06 (0.81 to 1.37)	0.85 (0.51 to 1.43)
PACIFIC	Antonia et al., 2018 (26)	0.68 (0.47 to 1.00)	0.62 (0.44 to 0.86)	0.76 (0.55 to 1.06)	N/A	N/A
CheckMate227	Hellmann et al., 2019 (22)	0.79 (0.65 to 0.96)	0.70 (0.55 to 0.89)	N/A	0.91 (0.70 to 1.19)	0.92 (0.57 to 1.48)

a Progression-free survival. CI = confidence interval; HR = hazard ratio; N/A = not applicable.

### Age

Of the experimental studies, 17 reported data across age groups (Table 3). Of the 11 that defined age as binary, 8 studies showed statistically significant improvements in OS or PFS with immunotherapy for patients aged younger than 65 years, compared with 3 studies for patients aged 65 years and older.

Six studies reported age as a trinomial variable (16,18,19, 21,22,25); 2 of which reported statistically significantly improved survival with immunotherapy for those aged younger than 65 years and those 65-75 years (Table 3) (19,21). One study reported improved survival only in patients aged younger than 65 years (22), and 1 did not report confidence intervals by age (18). No study reported statistically significant OS or PFS improvements with immunotherapy for patients aged older than 75 years.

### Smoking Status

Of the experimental studies, 15 reported hazard ratios stratified by smoking status (Table 4). Of the 12 studies that dichotomized smoking status, 6 had statistically significantly improved survival with immunotherapy for ever-smokers (13,19,21-23,26). Of these 6 studies, 2 also showed statistically significantly improved survival for never-smokers (13,26). Three RCTs grouped smoking as trinomial (11,12,20); 2 (KEYNOTE-042 and KEYNOTE-024) showed statistically significant OS or PFS improvements with immunotherapy for former smokers (11,12). No statistically significant differences were found for never- or current smokers.

**Table 4.** Association between survival and immunotherapy, stratified by smoking status in experimental studies (n = 15)

Clinical trial	Author, year	Immunotherapy vs no immunotherapy by smoking status				
		HR (95% CI)				
		All patients	Never	Ever	Former	Current
CheckMate 017	Brahmer et al., 2015 (19)	0.59 (0.44 to 0.79)	NR	0.59 (0.44 to 0.80)	N/A	N/A
CheckMate 026	Carbone et al., 2017 (20)	1.08 (0.87 to 1.34)	1.02 (0.54 to 1.93)	N/A	1.09 (0.84 to 1.42)	1.05 (0.63 to 1.74)
CheckMate 057	Borghaei et al., 2015 (21)	0.75 (0.62 to 0.91)	1.02 (0.64 to 1.61)	0.69 (0.56 to 0.86)	N/A	N/A
KEYNOTE-024^a^	Reck et al., 2016 (11)	0.50 (0.37 to 0.68)	0.90 (0.11 to 7.48)	N/A	0.47 (0.33 to 0.67)	0.68 (0.36 to 1.30)
KEYNOTE-042	Mok et al., 2019 (12)	0.81 (0.71 to 0.93)	1.00 (0.73 to 1.37)	N/A	0.71 (0.59 to 0.86)	0.95 (0.70 to 1.29)
KEYNOTE-189	Gandhi et al., 2018 (13)	0.49 (0.38 to 0.64)	0.23 (0.10 to 0.54)	0.54 (0.41 to 0.71)	N/A	N/A
POPLAR	Fehrenbacher et al., 2016 (27)	0.73 (0.53 to 0.99)	0.55 (0.24 to 1.25)	0.75 (0.54 to 1.04)	N/A	N/A
OAK	Rittmeyer et al., 2017 (23)	0.73 (0.62 to 0.87)	0.71 (0.47 to 1.08)	0.73 (0.61 to 0.88)	N/A	N/A
JAVELIN Lung 200	Barlesi et al., 2018 (24)	0.90 (0.73 to 1.12)	1.69 (0.97 to 2.95)	0.83 (0.66 to 1.04)	N/A	N/A
CheckMate227	Hellmann et al., 2019 (22)	0.79 (0.65 to 0.96)	1.23 (0.76 to 1.98)	0.77 (0.64 to 0.92)	N/A	N/A
PACIFIC	Antonia et al., 2018 (26)	0.68 (0.47 to 1.00)	0.35 (0.16 to 0.76)	0.72 (0.56 to 0.92)	N/A	N/A
IMpower130	West et al., 2019 (15)	0.79 (0.64 to 0.98)	0.55 (0.26 to 1.19)	0.81 (0.65 to 1.02)	N/A	N/A
IMpower131	Jotte et al., 2020 (16)	0.88 (0.73 to 1.05)	0.85 (0.43 to 1.68)	0.87 (0.72 to 1.05)	N/A	N/A
IMpower132	Nishio et al., 2020 (17)	0.86 (0.71 to 1.06)	0.78 (0.42 to 1.43)	0.89 (0.72 to 1.09)	N/A	N/A
IMpower150^a^	Socinski et al., 2018 (18)	0.62 (0.52 to 0.74)	0.8	0.58	N/A	N/A

a Progression-free survival. CI = confidence interval; HR = hazard ratio; N/A = not applicable.

### Sex

Of the experimental studies, 17 reported hazard ratios for immunotherapy, stratified by sex. There was statistically significant survival improvement with the addition of immunotherapy for males in 10 of 17 studies (10-14,19-23) and for females in 6 of 17 (Table 5) (10,13-15,23,26). Studies where only males saw improvements tested nivolumab (CheckMate 057; CheckMate 017) (19,21) and pembrolizumab (KEYNOTE-024; KEYNOTE-042) (11,12) monotherapy. The 2 studies in which only female patients saw improved OS [IMpower 130 (15); PACIFIC (26)] assessed combination therapies (atezolizumab and carboplatin combination and durvalumab and chemoradiotherapy combination, respectively).

**Table 5.** Association between survival and immunotherapy, stratified by sex in experimental studies (n = 18)

Clinical trial	Author, Year	Immunotherapy vs no immunotherapy by sex HR (95% CI)		
		All	Males	Females
CheckMate 017	Brahmer et al., 2015 (19)	0.59 (0.44 to 0.79)	0.57 (0.41 to 0.78)	0.67 (0.36 to 1.25)
CheckMate 057	Borghaei et al., 2015 (21)	0.75 (0.62 to 0.91)	0.73 (0.56 to 0.96)	0.78 (0.58 to 1.04)
KEYNOTE-010	Herbst et al., 2016 (10)	0.67 (0.56 to 0.80)	0.65 (0.52 to 0.81)	0.69 (0.51 to 0.94)
KEYNOTE-024^a^	Reck et al.,2016 (11)	0.50 (0.37 to 0.68)	0.39 (0.26 to 0.58)	0.75 (0.46 to 1.21)
CheckMate 026	Carbone et al., 2017 (20)	1.08 (0.87 to 1.34)	0.97 (0.74 to 1.26)	1.15 (0.79 to 1.66)
OAK, 2017	Rittmeyer et al., 2017 (23)	0.73 (0.62 to 0.87)	0.79 (0.64 to 0.97)	0.64 (0.49 to 0.85)
JAVELIN Lung 200	Barlesi et al., 2018 (24)	0.90 (0.73 to 1.12)	0.83 (0.64 to 1.08)	1.08 (0.74 to 1.59)
KEYNOTE-042	Mok et al., 2019 (12)	0.81 (0.71 to 0.93)	0.80 (0.68 to 0.94)	0.89 (0.68 to 1.17)
KEYNOTE-189	Gandhi et al., 2018 (13)	0.49 (0.38 to 0.64)	0.70 (0.50 to 0.99)	0.29 (0.19 to 0.44)
KEYNOTE-407	Paz-Ares et al., 2018 (14)	0.64 (0.49 to 0.85)	0.69 (0.51 to 0.94)	0.42 (0.22 to 0.81)
PACIFIC	Antonia et al., 2018 (26)	0.68 (0.47 to 1.00)	0.78 (0.59 to 1.03)	0.46 (0.30 to 0.73)
CheckMate 227	Hellmann et al., 2019 (22)	0.79 (0.65 to 0.96)	0.75 (0.61 to 0.93)	0.91 (0.69 to 1.21)
CA184-104	Govindan et al., 2017 (25)	0.91 (0.77 to 1.07)	0.85 (0.71 to 1.02)	1.33 (0.84 to 2.11)
IMpower130	West et al., 2019 (15)	0.79 (0.64 to 0.98)	0.87 (0.66 to 1.15)	0.66 (0.46 to 0.93)
IMpower131	Jotte et al., 2020 (16)	0.88 (0.73 to 1.05)	0.91 (0.75 to 1.12)	0.68 (0.44 to 1.04)
IMpower132	Nishio et al., 2020 (17)	0.86 (0.71 to 1.06)	0.93 (0.73 to 1.18)	0.76 (0.54 to 1.09)
IMpower150^a^	Socinski et al., 2018 (18)^b^	0.62 (0.52 to 0.74)	0.55	0.73

a Progression-free survival. CI = confidence interval; HR = hazard ratio.

^b^Socinski et al. did not report a 95% confidence interval for males and females.

### Race

Of the 5 RCTs that reported OS hazard ratios by race (16,17,24–26), only the PACIFIC study (durvalumab and chemoradiotherapy combination) (26) reported statistically significant improvement with immunotherapy. Asian and White patients derived similar benefit from immunotherapy, but the improvement was statistically significant only for White patients (26).

Two of the remaining studies reported hazard ratios for Asian, Black, and White patients (16,25), and 1 reported this data for only Asian and White patients (17). Distinctly, Barlesi et al. (24) reported race either as Hispanic or Latino, Japanese living in Japan, non-Hispanic or non-Latino, or Other. There were no statistically significant differences in OS in any of these studies (Supplementary Table 3, available online).

### Narrative Synthesis of Observational Studies

Of the observational studies where all patients received immunotherapy, 16 assessed at least one of age, smoking status, sex, or race. Six studies reported hazard ratios based on PFS, and 10 studies reported hazard ratios for OS (Table 2). Pembrolizumab and nivolumab (PD-1 inhibitors) were the most frequently studied immunotherapy agents. Both were used in all lines of treatment as monotherapies (5,33,39) and as part of combination therapy (31). Atezolizumab, ipilimumab, durvalumab, sintilimab, and tislelizumab were the other evaluated immunotherapy regimens.

### Age

Of the 13 studies reporting hazard ratios for age, 4 reported statistically significantly better OS or PFS for younger patients compared with older patients (Table 6) (30–32,41). A Dutch national database study (n = 2302) of patients with stage IV NSCLC in all lines of treatment found no statistically significant difference in OS by age (29). However, in a US-based study (n = 5807) of stage IV NSCLC patients who received first-line immunotherapy treatment, those aged 18-59 years had longer overall survival than older patients (41).

**Table 6.** Association between survival after immunotherapy and age in observational studies (n = 13)

	Age, y				
Study	Reference	Comparison	Univariate HR (95% CI)	Multivariate HR (95% CI)	Adjustments
Chen et al., 2020 (28)^a^	<65	≥65	1.20 (0.73 to1.95)	N/A	N/A
Smit et al., 2020 (29)	28-74	75-88	0.84 (0.66 to 1.08)	N/A	N/A
Lichtenstein et al., 2019 (30)	<60	60-69	N/A	0.76 (0.46-1.25)	CCI, initial stage, sex, ECOG PS
		70-79	N/A	0.93 (0.57-1.51)	
		≥80	N/A	2.74 (1.42-5.25)	
Huang et al., 2020 (32)	<65	≥ 5	3.88 (1.69 to 8.92)	5.45 (1.98-14.98)	Metastasis, NLR C4, CEA, irAE, line of therapy, response to therapy
Prelaj et al., 2019 (33)^a^	<70	≥70	N/A	1.20 (0.85-1.69)	Sex, smoking, ECOG PS, histology, metastasis
Elkrief et al., 2020 (34)	<70	≥70	N/A	0.78 (0.56 to 1.08)	Sex, smoking, ECOG PS, histology, stage, line of treatment, anti-PD-1 agent
Anouti et al., 2020 (36)	<65	≥65	1.00 (0.97 to 1.03)	N/A	N/A
Adachi et al., 2019 (37)^a^	<70	≥70	1.09 (0.85 to 1.39)	N/A	N/A
Ahn et al., 2019 (38)	<75	≥75	0.95 (0.54 to 1.69)	0.71 (0.34 to 1.50)	Sex, smoking, prior treatment lines, mutations, brain and liver metastasis, PD-L1 expression level
Lin et al., 2018 (39)	<65	≥65	1.32 (0.70 to 2.49)	0.70 (0.35 to 1.42)	Sex, smoking, ECOG PS, brain metastasis, EGFR
Ng et al., 2018 (40)^a^	<65	≥65	0.75 (0.47 to 1.21)	N/A	N/A
Foster et al., 2019 (41)	18-59	60-69	N/A	1.08 (1.01 to 1.17)	NR
		70-79	N/A	1.14 (1.05 to 1.24)	
		≥80	N/A	1.26 (1.09 to 1.46)	
Yang et al., 2020 (31)^a^	<60	≥60	N/A	2.02 (1.30 to 3.14)	NR

a Progression-free survival. CCI = Charlson comorbidity index; CEA = carotid endarterectomy; CI = confidence interval; ECOG PS = Eastern Cooperative Oncology Group Performance Status; EGFR = epidermal growth factor receptor; HR = hazard ratio; irAE = immune-related adverse events; N/A = not applicable; NLR = neutrophil-to-lymphocyte ratio; NR = not reported.

A study of 61 hospitalized patients in China treated with pembrolizumab, nivolumab, atezolizumab, or ipilimumab (28% first-line; 72% second-line and later) showed statistically significantly worse survival for patients aged 65 years or older compared with those aged younger than 65 years, before and after adjustments for metastasis, adverse effects, line of therapy, and other biomarkers (32). In a multivariate model, Lichtenstein et al. (30) found immunotherapy patients aged older than 80 years had worse OS than those aged younger than 60 years. However, no statistically significant differences in OS were observed for patients in other age groups.

### Smoking Status

Of 13 studies, 8 reported statistically significant OS or PFS differences (either univariate or multivariate) for smoking status. Seven showed that among immunotherapy recipients, former and current smokers had statistically significantly better OS and PFS than never-smokers (29,33,35,37,39,40,42). Distinctly, only 1 study (n = 101) of stage III-IV NSCLC patients receiving third-line, immunotherapy with anlotinib combination therapy showed statistically better PFS for never-smokers relative to ever-smokers (Table 7) (31).

**Table 7.** Association between survival after immunotherapy and smoking status in observational studies (n = 13)

	Smoking status				
Author, year	Reference	Comparison	Univariate HR (95% CI)	Multlivariate HR (95% CI)	Adjustments
Chen et al., 2020 (28)^a^	Smoker	Nonsmoker	1.23 (0.76 to 1.96)	N/A	N/A
Smit et al., 2020 (29)	Smoker	Nonsmoker	1.28 (1.00 to 1.63)	N/A	N/A
Yang et al., 2020 (31)^a^	Smoker	Nonsmoker	N/A	0.35 (0.21 to 0.59)	NR
Song et al., 2020 (42)^a^	Smoking index ≥ 400	Smoking Index <400	N/A	2.7 (1.37 to 5.26)	PD-L1 expression, NLR
Huang et al., 2020 (32)	Smoker	Nonsmoker	0.94 (0.47 to 1.86)	N/A	N/A
Prelaj et al., 2019 (33)^a^	≥ 40 packs/year	<40 packs/year	N/A	1.39 (1.02 to 1.92)	Age, sex, ECOG PS, histology, metastasis
Elkrief et al., 2020 (34)	Smoker	Nonsmoker	N/A	0.84 (0.51 to 1.38)	Sex, ECOG PS, histology, stage, line of treatment, anti-PD-1 agent
Kano et al., 2020 (35)	Former/Current smoker	Never smoker	1.28 (0.94 to 1.73)	1.82 (1.03 to 3.09)	NR
Anouti et al., 2020 (36)	Current smoker	Never smoker	0.59 (0.12 to 2.78)	N/A	N/A
Adachi et al., 2019 (37)^a^	Former/Current smoker	Never smoker	1.42 (1.05 to 1.93)	1.68 (1.16 to 2.43)	ECOG PS, driver mutation, LDH, CRP, ALB, NLR, ALI, liver and brain metastasis, pleural effusion, steroid use
Ahn et al., 2019 (38)	Former/Current smoker	Never smoker	1.02 (0.65 to 1.62)	0.88 (0.26 to 2.99)	Age, sex, prior treatment lines, mutational status, brain and liver metastasis, PD-L1 expression
Lin et al., 2018 (39)	smoker	Nonsmoker	1.39 (0.73 to 2.63)	2.27 (1.10 to 4.67)	Age, sex, ECOG PS ≥ 2, brain metastasis, EGFR

a Progression-free survival. ALB = albumin blood; ALI = acute lung injury; CI = confidence interval; CRP = C-reactive protein; ECOG PS = Eastern Cooperative Oncology Group Performance Status; EGFR = epidermal growth factor receptor; HR = hazard ratio; LDH = lactate dehydrogenase; N/A = not applicable; NLR = neutrophil-to-lymphocyte ratio; NR = not reported.

The 2 largest studies examining multiple immunotherapy agents showed statistically significantly better survival for smokers than nonsmokers (29,35). In Smit and colleagues’ (29) analysis of Dutch patients (n = 2302), nonsmokers had statistically significantly worse survival than smokers. In the Japanese Okayama Lung Cancer Study of NSCLC patients in all lines of treatment with metastatic or recurrent disease, univariate results were not statistically significant. However, after adjustments, never-smokers had statistically significantly worse survival than former and current smokers (35).

### Sex

Of 14 studies reporting on sex, 12 found no statistically significant differences in OS or PFS in males compared with females treated with immunotherapy (Table 8). However, the largest study (n = 5807) of patients receiving first-line immunotherapy found females had better survival than males (41). Another retrospective analysis of patients (n = 257) treated with single-agent pembrolizumab, nivolumab, or atezolizumab showed statistically significantly better OS for females than males only in the adjusted model (5).

**Table 8.** Association between survival after immunotherapy and sex in observational studies (n = 14)

	Sex				
Author, year	Reference	Comparison	Univariate HR (95% CI)	Multivariate HR (95% CI)	Adjustments
Chen et al., 2020 (28)^a^	Female	Male	1.05 (0.63 to 1.75)	N/A	N/A
Yang et al., 2020 (31)^a^	Female	Male	N/A	1.28 (0.79 to 2.09)	NR
Huang et al., 2020 (32)	Female	Male	1.12 (0.57 to 2.23)	N/A	N/A
Nazha et al., 2020 (5)	Female	Male	1.43 (0.97 to 2.08)	8.33 (2.5 to 25)	NR
Prelaj et al., 2019 (33)^a^	Female	Male	N/A	1.06 (0.76 to 1.47)	Age, smoking, ECOG PS, histology, metastasis
Elkrief et al., 2020 (34)	Female	Male	N/A	0.81 (0.58 to 1.12)	Smoking, ECOG PS, histology, stage, line of treatment, anti-PD-1 agent
Kano et al., 2020 (35)	Female	Male	0.95 (0.71 to 1.27)	1.57 (0.94 to 2.60)	N/A
Anouti et al., 2020 (36)	Female	Male	1.12 (0.58 to 2.13)	N/A	N/A
Adachi et al., 2019 (37)^a^	Female	Male	0.96 (0.74 to 1.27)	N/A	N/A
Ahn et al., 2019 (38)	Female	Male	1.17 (0.75 to 1.82)	0.527 (0.15 to 1.85)	Age, smoking, prior treatment, mutational status, brain and liver metastasis, PD-L1 expression
Lin et al., 2018 (39)	Female	Male	0.71 (0.37 to 1.34)	N/A	N/A
Ng et al., 2018 (40)^a^	Female	Male	1.36 (0.86 to 2.16)	N/A	N/A
Foster et al., 2019 (41)	Female	Male	N/A	1.26 (1.19 to 1.33)	NR
Lichtenstein et al., 2019 (30)	Female	Male	N/A	1.13 (0.83 to 1.54)	CCI, initial cancer stage, ECOG PS

a Progression-free survival. CCI = Charlson comorbidity index; CI = confidence interval; HR = hazard ratio; N/A = not applicable; NR = not reported; ECOG PS = Eastern Cooperative Oncology Group Performance Status.

### Race

Only 3 observational studies assessed the association between survival and race, among immunotherapy patients (Supplementary Table 4, available online) (5,40,41). Foster et al. (41) found non-White patients had statistically significantly better OS than White patients.

Nazha et al. (5) observed no differences in OS by race. A univariate analysis of patients in Colorado and Shanghai with targetable driver mutations in all treatment lines showed better survival for non-Asian compared with Asian patients, but the difference was not statistically significant in the multivariate analysis (40).

## Discussion

This systematic review was performed to evaluate whether age, smoking status, sex, and race modify the effectiveness of immunotherapy in late-stage NSCLC and to assess variability in survival among patients who received immunotherapy. We reviewed 18 experimental studies comparing immunotherapy and nonimmunotherapy groups (n = 6534 and 11 192, respectively) and 16 observational studies of 9073 patients who received immunotherapy. Because of study design differences, results from experimental and observational studies are complementary, rather than comparable. Experimental hazard ratios reflect benefit from the addition of immunotherapy (compared with those without) and allow us to assess factors that may modify immunotherapy efficacy. Immunotherapy was universal across patients in observational studies, which focus on directly comparing survival among patient subgroups. As such, observational studies may provide insight into whether differential efficacy of immunotherapy contributes to survival disparities.

Experimental studies generally suggested that younger patients derive more benefit from immunotherapy than their older counterparts. In particular, survival outcomes of patients aged 75 years and older tended to be less favorable, and trends were less consistent than for patients aged younger than 75 years. For subgroups of patients aged younger than 65 years, all experimental studies except CheckMate 026 (20) showed a trend toward improved survival with receipt of immunotherapy, even if the improvement in survival was not statistically significant. Likewise, with the exception of the CA184-104 trial (25), all subgroups of patients aged 65-75 years trended toward receiving benefit from immunotherapy. This is consistent with research showing older adults are more susceptible to the onset of immunosenescence and reduced intrinsic immunity (43). Increased age is also linked to a longer period of carcinogenesis as well as increased vulnerability and sensitization of cells to environmental carcinogens (44,45).

However, when directly comparing survival by age group, the majority of observational studies found no statistically significant difference. This is somewhat paradoxical, as younger lung cancer patients generally survive longer (44,46). The 2 largest observational studies had inconsistent findings, although they assessed different treatment lines. This may suggest that the marginal benefit of immunotherapy by age may differ across treatment lines. More uniform reporting of outcomes would be needed to address this hypothesis.

For smoking status, experimental studies more frequently showed increased survival benefit in smokers compared with nonsmokers. Likewise, most observational studies reported improved survival for current or former smokers but not never-smokers. These results are consistent with previous reviews and may reflect the fact that smokers tend to have more tumor mutations, corresponding to greater immunogenicity and a higher likelihood of immune cell recognition of tumor cells (47). For instance, whole-genome sequencing revealed smokers had a tenfold higher mutation frequency than never-smokers (48). Considering nonsmokers tend to have fewer comorbidities and are generally healthier than smokers (49), this suggests nonsmokers may not be ideal candidates for immunotherapy, especially in the absence of a high tumor mutational burden.

Two clinical trials—KEYNOTE-189 (13) and PACIFIC (26)—yielded statistically significant improvement in OS for never-smokers, and more so than in ever-smokers. These findings differ from the majority of included studies and may be partially attributable to the unique characteristics of the trials. KEYNOTE-189 was the only trial to have an immunotherapy intervention arm with both pembrolizumab and pemetrexed (13). The PACIFIC trial, which enrolled patients in second-line and later treatment lines, was the only included trial to enroll patients with unresectable stage III NSCLC or to assess either durvalumab or chemoradiotherapy (26). As such, combination therapies like pembrolizumab and pemetrexed as well as durvalumab and chemoradiotherapy ought to be further examined as potential options for immunotherapy treatment of patients who may otherwise be poor responders.

Findings around sex were mixed, reflecting the ongoing debate in the current literature (50). Among the 17 experimental studies reporting data stratified by sex, 10 reported statistically significant improvements with the addition of immunotherapy for males, compared with only 6 for females. However, every experimental study trended toward survival improvement for males with the addition of immunotherapy, even when results did not rise to the level of statistical significance. Results for female patients were less consistent, although trends from most studies did suggest some potential benefit. Notably, the KEYNOTE-189 trial, which uniquely evaluated pembrolizumab–pemetrexed combination therapy, reported especially high survival gains for females (HR = 0.29) that exceeded those seen in male patients (HR = 0.70) (13). Given the different trends for KEYNOTE-189 with regard to both smoking status and sex, this combination may warrant future examination. Most observational studies found no statistically significant difference in survival by sex, although the largest found statistically significantly better survival for females, compared with males, consistent with previous research (51). Although there is uncertainty, one interpretation of these findings is that even if males derive more benefit from immunotherapy than females, the increased benefit is insufficient to overcome the existing female survival advantage (41,51,52). Although a specific mechanism for NSCLC is still unclear, sex hormones such as estrogen and testosterone, which have immunogenic and immunosuppressive properties, respectively, are known to modulate gene expression, immune system agents, and treatment-related adverse effects (53,54). Additional research is needed to further examine moderating factors that may favor positive prognoses by sex. Moreover, only 1 clinical trial had approximately equal enrollment of males and females (21): the rest were at least 59% male, with 6 trials enrolling 70% males or more. Therefore, these results reinforce the need for proper representation of females in clinical trials to better evaluate how sex may affect survival and response to immunotherapy.

Only 5 experimental studies and 3 observational studies provided race information. More data is needed on outcomes according to race in clinical trials, considering recent evidence of differential tumor mutation burden (55,56). Trials reporting outcomes by race were severely underpowered because of their disproportionate underenrollment of non-White patients. Despite representing 13% of the US population, Black patients comprised no more than 4% of any clinical trial. Although in Foster and colleagues’ study (41), non-White patients survived longer than White patients, the classification of non-White makes interpretation difficult and further highlights the need for more diverse, inclusive clinical trials with details on patients’ ethnic and racial backgrounds.

This review highlights several gaps in current research on immunotherapy efficacy. There was uneven reporting of univariate and multivariate hazard ratios among the included observational studies. Even when multivariate hazard ratios were reported, not all studies specified adjustment variables, and no 2 studies used the same set of covariate adjustments. To minimize some of this variation, specific covariates that will be adjusted for in multivariate hazard ratios or adjusted risk calculations should be chosen and reported a priori. Establishing a consensus around adjustments will facilitate comparisons between different studies. Lastly, more frequent reporting of interaction terms (ie, age/sex/smoking status/race*immunotherapy) in observational studies may allow for more direct comparison with experimental studies and provide more information about effectiveness outside of a clinical trial setting, where male, younger, and healthier patients are frequently overenrolled (57). As such, greater consistency in methodology and reporting of risk calculations can further the understanding of clinical confounders that may contribute to differential survival outcomes, and ultimately, help tailor patient therapies.

###  

To our knowledge, this systematic review is the first to aggregate evidence across both experimental and observational studies and analyze outcomes with respect to multiple personal characteristics, including race. Although head-to-head comparisons with previous reviews are difficult because of differences in inclusion and exclusion criteria and patient populations, many of our findings from observational studies pertaining to age and smoking status align with those in previous reviews of experimental studies (47,58-60).

This review is not without limitations. Because of the heterogeneity in patient populations, immunotherapies administered, and uneven reporting of outcomes, meta-analysis was not possible. Moreover, although there were a couple of national database studies that met all inclusion criteria, most observational studies were retrospective analyses of patient cohorts at individual hospitals. Therefore, some studies may have limited generalizability to the broader patient population receiving immunotherapy. Publication bias toward negative findings is also a concern as both observational studies and clinical trials that fail to identify statistically significant differences or associations are less likely to be published.

Overall, evidence is mixed around which personal characteristics may be associated with increased benefit from immunotherapy treatment in advanced NSCLC. In aggregate, the findings from this review confirm those of previous reviews and individual studies, which suggest immunotherapy may increase survival in younger patients, smokers, and males.

We also highlight gaps in the literature, as very few experimental and observational studies have reported outcomes by race and those which have are severely underpowered. Further research into the moderating effects of race on immunotherapy efficacy can fill this gap while potentially creating more opportunities to better target immunotherapies and improve clinical care. To further explore disparities in immunotherapy effectiveness, future research should also include patient-level analyses of national registries (41). This will enable direct adjustment for individual characteristics and assessment of treatment interactions in patients more representative of the general late-stage NSCLC population.

## Funding

Not applicable.

## Notes

**Role of the funder:** Not applicable.

**Disclosures:** All authors have no conflicts of interest, disclosures, to report for this work.

**Author contributions:** Conceptualization: KP, NA, ET. Formal analysis: KP, NA, ST, ET. Methodology: KP, NA, ET. Writing—original draft: KP, NA, ST, ET. Writing—review and editing: KP, NA, ST, ET.

**Acknowledgements:** We would like to thank Cindy Patippe for her help with quality review.

**Prior** **presentations:** An abstract based on this work was presented as a E-poster abstract at the 2021 International Association for the Study of Lung Cancer (IASLC) World Conference on Lung Cancer held on September 8, 2021.

## Data Availability

The data underlying this article are available in the article and in its online supplementary material.

## Supplementary Material

pkac015_Supplementary_Data
