# Supplementary material for: Association of Personal Characteristics and Effectiveness of Immunotherapy in Late-Stage Non-Small Cell Lung Cancer: A Systematic Review
Source: JNCI Cancer Spectr. 2022 Feb 17;6(2):pkac015. doi: 10.1093/jncics/pkac015 (PMC8935137; doi:10.1093/jncics/pkac015)
Supplement: pkac015_Supplementary_Data [file pkac015_supplementary_data.pdf]

<u>Supplementary Materials</u>

Supplementary Tables

Supplementary Table 1: National Institutes of Health Assessment of Controlled Intervention Studies Scoring

Study	Q1: Study described as RCT?	Q2: Randomization method?	Q3: Allocation Concealment?	Q4: Participants & Provider Blinded?	Q5: Outcome assessors blinded to group assignment?	Q6: Similar groups at baseline?	Q7: Dropout <20%?	Q8: Differential Dropout between groups <15%?	Q9: Adherence to protocol?	Q10: Absence of other/different interventions between groups?	Q11: Outcome measurements valid/consistent?	Q12: Sufficient n/ power to detect differences in main outcome between groups with 80% power?	Q13: Pre-specified outcomes/subgroup analysis?	Q14: Analyzed in group originally assigned (i.e. ITT)?	Quality
Antonia, et al. 2018 (1)	Y	Y	Y	Y	Y	Y	Y	Y	Y	Y	Y	Y	Y	Y	14/14
Barlesi, et al. 2018 (2)	Y	Y	N	N	N	Y	Y	Y	Y	Y	Y	Y	Y	Y	11/14
Borghaei, et al. 2015 (3)	Y	Y	Y	N	N	Y	Y	Y	Y	Y	Y	Y	Y	NR	11/14
Brahmer, et al. 2015 (4)	Y	Y	Y	N	N	Y	Y	Y	Y	Y	Y	Y	Y	Y	12/14
Carbone, et al. 2017 (5)	Y	Y	Y	N	Y	Y	Y	Y	Y	Y	Y	Y	N	NR	10/14
Fehrenbacher, et al. 2016 (6)	Y	Y	Y	N	N	Y	Y	Y	Y	Y	Y	Y	Y	Y	12/14

Gandhi, et al. 2018 (7)	Y	Y	Y	Y	Y	Y	Y	Y	Y	Y	Y	Y	Y	Y	14/14
Govindan, et al. 2017 (8)	Y	Y	Y	Y	Y	Y	Y	Y	NR	Y	Y	Y	Y	Y	13/14
Hellmann, et al. 2019 (9)	Y	Y	Y	N	Y	Y	Y	Y	Y	Y	Y	Y	Y	NR	12/14
Herbst, et al. 2016 (10)	Y	Y	Y	N	N	Y	Y	Y	Y	Y	Y	Y	Y	Y	12/14
Jotte, et al. 2020 (11)	Y	Y	NR	N	N	Y	Y	Y	Y	Y	Y	NR	Y	Y	10/14
Mok, et al. 2019 (12)	Y	Y	Y	N	N	Y	Y	Y	Y	Y	Y	Y	Y	Y	12/14
Nishio, et al. 2020 (13)	Y	Y	Y	N	N	Y	Y	Y	Y	Y	Y	Y	Y	Y	12/14
Paz Ares, et al. 2018 (14)	Y	Y	Y	Y	Y	Y	Y	Y	Y	Y	Y	Y	Y	Y	14/14
Reck, et al. 2016 (15)	Y	Y	Y	N	Y	Y	Y	Y	Y	Y	Y	Y	Y	Y	13/14
Rittmeyer, et al. 2017 (16)	Y	Y	N	N	N	Y	Y	Y	Y	Y	Y	Y	Y	Y	11/14
Socinski, et al. 2018 (17)	Y	Y	NR	N	N	Y	Y	Y	Y	Y	Y	Y	Y	Y	11/14
West, et al. 2019 (18)	Y	Y	N	N	N	Y	Y	Y	Y	Y	Y	N	Y	Y	10/14

[a] National Institutes of Health (2021a). Quality Assessment of Controlled Intervention Studies. Available online at: https://www.nhlbi.nih.gov/health-topics/study-quality-assessment-tools (Accessed 4/14/21). Y=Yes, N=No, NR=Not reported, ITT= Intention-to-treat, RCT=Randomized-control trial.

Supplementary Table 2: National Institutes of Health Assessment for Observational Cohort and Cross-Sectional Studies [a]

Study	Q1: Research objective clearly stated?	Q2: Study population specified?	Q3: 50% participation rate among eligible?	Q4: Recruitment from similar populations/ times?	Q5: Justification for n/power/ effect estimate?	Q6: Exposures measured before outcomes being measured?	Q7: Sufficient timeframe?	Q8: Different levels of exposure measured?	Q9: Exposure measures consistent across all participants?	Q10: Exposure assessed more than once?	Q11: Valid/ consistently implemented outcome measures	Q12: Outcome assessor blinded?	Q13: <20% loss to follow up	Q14: Measurement of/adjustment for confounding variables?	Quality
Adachi, et al. 2019 (19)	Y	Y	Y	Y	N	Y	Y	NA	Y	Y	Y	NA	NA	Y	10/14
Ahn, et al. 2019 (20)	Y	Y	Y	Y	N	Y	Y	NA	Y	Y	Y	NA	NA	Y	10/14
Anouti, et al. 2020 (21)	Y	Y	Y	Y	N	Y	Y	NA	Y	Y	Y	NA	NA	Y	10/14
Chen, et al. 2020 (22)	Y	Y	Y	Y	N	Y	Y	NA	Y	Y	Y	NA	NA	Y	10/14
Elkrief, et al. 2020 (23)	Y	Y	Y	Y	N	Y	Y	NA	Y	Y	Y	NA	NA	Y	10/14
Foster, et al. 2019 (24)	Y	Y	Y	Y	N	Y	Y	NA	Y	Y	Y	NA	NA	Y	10/14
Huang, et al. 2020 (25)	Y	Y	Y	Y	N	Y	Y	NA	Y	Y	Y	NA	NA	Y	10/14
Kano, et al. 2020 (26)	Y	Y	Y	Y	N	Y	Y	NA	Y	Y	Y	NA	NA	Y	10/14
Lichtenstein, et al. 2020 (27)	Y	Y	Y	Y	N	Y	Y	NA	Y	Y	Y	NA	NA	Y	10/14
Lin, et al. 2018 (28)	Y	Y	Y	Y	N	Y	Y	NA	Y	Y	Y	NA	NA	Y	10/14
Nazha, et al. 2020 (29)	Y	Y	Y	Y	N	Y	Y	NA	Y	Y	Y	NA	NA	Y	10/14
Ng, et al. 2018 (30)	Y	Y	Y	Y	N	Y	Y	NA	Y	Y	Y	NA	NA	Y	10/14
Prelaj, et al. 2019 (31)	Y	Y	Y	Y	N	Y	Y	NA	Y	Y	Y	NA	NA	Y	10/14

Smit, et al. 2020 (32)	Y	Y	Y	Y	N	Y	Y	NA	Y	Y	Y	NA	NA	Y	10/14
Song, et al. 2020 (33)	Y	Y	Y	Y	N	Y	Y	NA	Y	Y	Y	NA	NA	Y	10/14
Yang, et al. 2020 (34)	Y	Y	Y	Y	N	Y	Y	NA	Y	Y	Y	NA	NA	Y	10/14

[a] National Institutes of Health (2021b). Quality Assessment Tool for Observational Cohort and Cross-Sectional Studies. Available online at: https://www.nhlbi.nih.gov/health-topics/study-quality-assessment-tools (Accessed 4/14/21). Y=Yes, N=No, NA=Not Applicable, ITT= Intention-to-treat, RCT=Randomized-control trial.

Supplementary Table 3: Association between survival and immunotherapy stratified by race in experimental studies (n=5)

		Immunotherapy vs. No Immunotherapy by Race HR (95% CI)				
Clinical Trial	Author, Year	All	Asian	Black	"OtherRaces”	White
CA184-104	Govindan, et al. 2017 (8)	0.91 (0.77, 1.07)	0.73 (0.51, 1.03)	0.99 (0.16, 6.12)	N/A	0.94 (0.78, 1.15)
PACIFIC	Antonia, et al. 2018 (1)	0.68 (0.47, 1.00)	0.62 (0.38, 1.01)	N/A	N/A	0.71 (0.54, 0.93)
IMpower 131	Jotte, et al. 2020 (11)	0.88 (0.73, 1.05)	1.31 (0.70, 2.42)	0.67 (0.12, 3.67)	N/A	0.84 (0.69, 1.02)
IMpower132	Nishio, et al. 2020 (13)	0.86 (0.71, 1.06)	0.73 (0.46, 1.15)	N/A	N/A	0.90 (0.72, 1.14)
JAVELIN Lung 200	Barlesi, et al. 2018 (2)	0.90 (0.73, 1.12)	0.95 (0.56, 1.61)[c]	0.96 (0.52, 1.77)[b]	0.89 (0.71, 1.13)[d]	0.87 (0.68, 1.11)[a]

[a] For this study, this category was designated Non-Hispanic/Non-Latino. HR = hazard ratio; N/A = not applicable.

[b] For this study, this category was designated Hispanic or Latino.

[c] For this study, this category was designated Japanese Living in Japan.

[d] For this study, “Other Race” includes patients who were not defined in the categories listed in footnotes b-c. No further breakdown of these categories was provided in the study

Supplementary Table 4: Association between survival after immunotherapy and race in observational studies (n=3)

	Race				
Author, Year	Reference	Comparison	Univariate HR (95% CI)	Mutlivariate HR (95% CI)	Adjustments
Foster, et al. 2019 (24)	White	Non-White	N/A	0.89 (0.82, 0.98)	NR
Nazha, et al. 2020 (29)	White/Caucasian	Black/African American<br>Other	0.9 (0.59, 1.37)<br>0.33 (0.10, 1.04)	N/A	N/A
Ng, et al. 2018 (30)[a]	Asian	Non-Asian	0.60 (0.36, 1.00)	2.02 (0.49, 8.26)	NR

[a] Progression free survival (PFS). HR = hazard ratio; N/A = not applicable; NR = not reported

Supplementary Figure

Supplementary Figure 1: PRISMA flowchart of search results. HR = hazard ratio.

NSCLC Search Results: TI=(Immuno* OR CHECK* OR PD-1 OR PD-L1 OR *mab OR cyramza) AND TI=(NSCLC OR "non-small cell lung cancer" OR "non small cell lung cancer") AND TS=(("sex" OR "gender" OR "smok*" OR "rac*" OR "ethnic*" OR "age" OR "Comorb*" OR "soci*" OR "socioec*"))

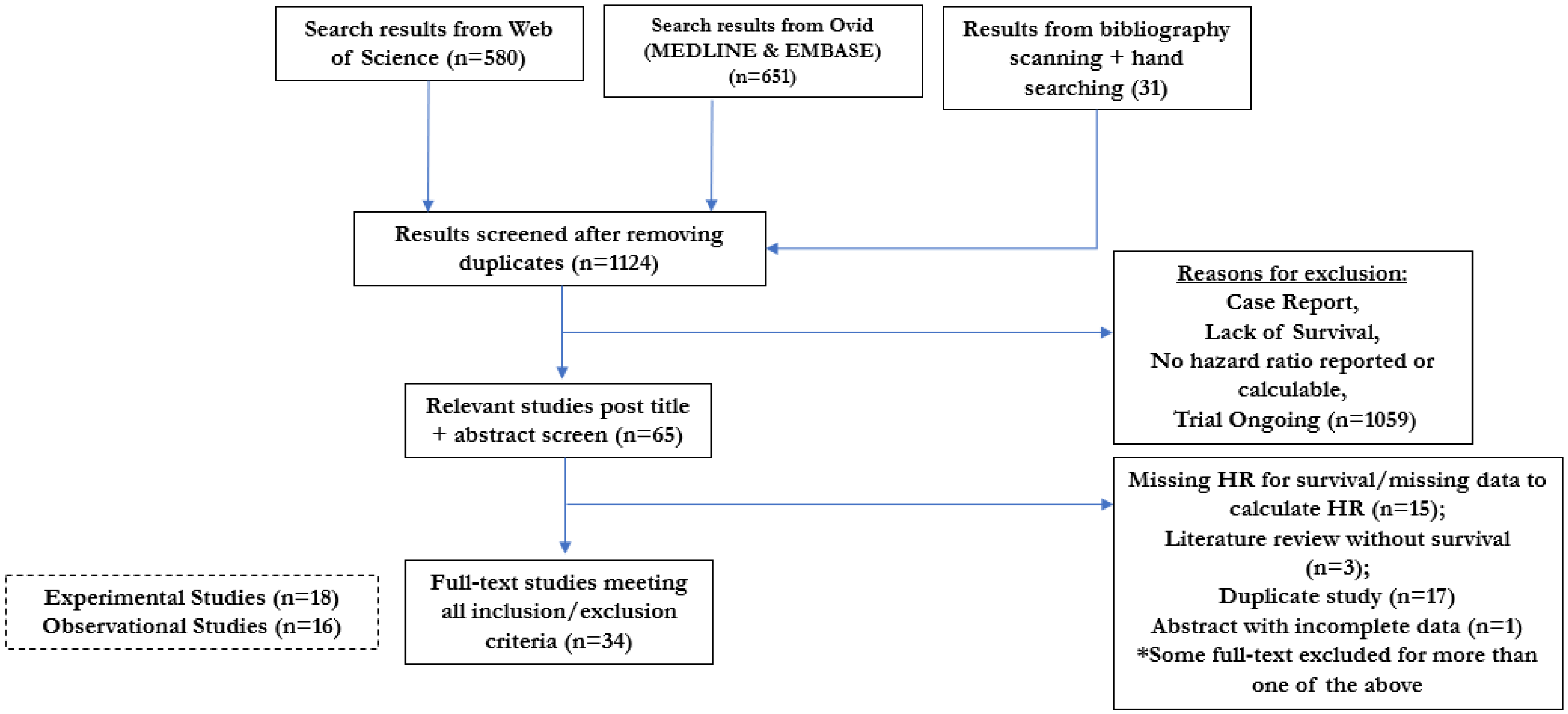

References:

1. Antonia SJ, Villegas A, Daniel D, et al. Overall Survival with Durvalumab after Chemoradiotherapy in Stage III NSCLC. 2018;379(24):2342 2350.

2. Barlesi F, Vansteenkiste J, Spigel D, et al. Avelumab versus docetaxel in patients with platinum-treated advanced non-small-cell lung cancer (JAVELIN Lung 200): an open-label, randomised, phase 3 study. *Lancet Oncol*. 2018;19(11):1468-1479.

3. Borghaei H, Paz-Ares L, Horn L, et al. Nivolumab versus Docetaxel in Advanced Nonsquamous Non–Small-Cell Lung Cancer. 2015;373(17):1627-1639.

4. Brahmer J, Reckamp KL, Baas P, et al. Nivolumab versus Docetaxel in Advanced Squamous-Cell Non–Small-Cell Lung Cancer. 2015;373(2):123-135.

5. Carbone DP, Reck M, Paz-Ares L, et al. First-Line Nivolumab in Stage IV or Recurrent Non–Small-Cell Lung Cancer. 2017;376(25):2415-2426.

6. Fehrenbacher L, Spira A, Ballinger M, et al. Atezolizumab versus docetaxel for patients with previously treated non-small-cell lung cancer (POPLAR): a multicentre, open-label, phase 2 randomised controlled trial. *Lancet.* 2016;387(10030):1837-1846.

7. Gandhi L, Rodríguez-Abreu D, Gadgeel S, et al. Pembrolizumab plus Chemotherapy in Metastatic Non–Small-Cell Lung Cancer. 2018;378(22):2078-2092.

8. Govindan R, Szczesna A, Ahn MJ, et al. Phase III Trial of Ipilimumab Combined With

Paclitaxel and Carboplatin in Advanced Squamous Non-Small-Cell Lung Cancer. *J Clin Oncol*. 2017;35(30):3449-3457.

9. Hellmann MD, Paz-Ares L, Bernabe Caro R, et al. Nivolumab plus Ipilimumab in Advanced Non–Small-Cell Lung Cancer. 2019;381(21):2020-2031.

10. Herbst RS, Baas P, Kim DW, et al. Pembrolizumab versus docetaxel for previously treated, PD-L1-positive, advanced non-small-cell lung cancer (KEYNOTE-010): a randomised controlled trial. *Lancet*. 2016;387(10027):1540-1550.

11. Jotte R, Cappuzzo F, Vynnychenko I, et al. Atezolizumab in Combination With Carboplatin and Nab-Paclitaxel in Advanced Squamous NSCLC (IMpower131): Results From a Randomized Phase III Trial. *J Thorac Oncol*. 2020;15(8):1351-1360.

12. Mok TSK, Wu YL, Kudaba I, et al. Pembrolizumab versus chemotherapy for previously untreated, PD-L1-expressing, locally advanced or metastatic non-small-cell lung cancer (KEYNOTE-042): a randomised, open-label, controlled, phase 3 trial. *Lancet*. 2019;393(10183):1819-1830.

13. Nishio M, Barlesi F, West H, et al. Atezolizumab Plus Chemotherapy for First-Line Treatment of Nonsquamous NSCLC: Results From the Randomized Phase 3 IMpower132 Trial. *J Thorac Oncol*. 2021;16(4):653-664.

14. Paz-Ares L, Luft A, Vicente D, et al. Pembrolizumab plus Chemotherapy for Squamous Non–Small-Cell Lung Cancer. 2018;379(21):2040-2051.

15. Reck M, Rodríguez-Abreu D, Robinson AG, et al. Pembrolizumab versus Chemotherapy for PD-L1–Positive Non–Small-Cell Lung Cancer. 2016;375(19):1823-1833.

16. Rittmeyer A, Barlesi F, Waterkamp D, et al. Atezolizumab versus docetaxel in patients with previously treated non-small-cell lung cancer (OAK): a phase 3, open-label, multicentre randomised controlled trial. *Lancet*. 2017;389(10066):255-265.

17. Socinski MA, Jotte RM, Cappuzzo F, et al. Atezolizumab for First-Line Treatment of Metastatic Nonsquamous NSCLC. 2018;378(24):2288-2301.18. West H, McCleod M, Hussein M, et al. Atezolizumab in combination with carboplatin

plus nab-paclitaxel chemotherapy compared with chemotherapy alone as first-line treatment for metastatic non-squamous non-small-cell lung cancer (IMpower130): a multicentre, randomised, open-label, phase 3 trial. *Lancet Oncol*. 2019;20(7):924-937.

19. Adachi Y, Tamiya A, Taniguchi Y, et al. Predictive factors for progression-free survival in non-small cell lung cancer patients receiving nivolumab based on performance status. *Cancer Med.* 2020;9(4):1383-1391.

20. Ahn BC, Pyo KH, Xin CF, et al. Comprehensive analysis of the characteristics and treatment outcomes of patients with non-small cell lung cancer treated with anti-PD-1 therapy in real-world practice. *J Cancer Res Clin Oncol.* 2019;145(6):1613-1623.

21. Anouti B, Althouse S, Durm G, Hanna N. Prognostic Variables Associated With Improved Outcomes in Patients With Stage III NSCLC

Treated With Chemoradiation Followed by Consolidation Pembrolizumab: A Subset Analysis of a Phase II Study From the Hoosier Cancer Research Network LUN 14-179. *Clin Lung Cancer*. 2020;21(3):288-293.

22. Chen M, Li Q, Xu Y, et al. Immunotherapy as second-line treatment and beyond for non-small cell lung cancer in a single center of China: Outcomes, toxicities, and clinical predictive factors from a real-world retrospective analysis. *Thorac Cancer*. 2020;11(7):1955-1962. doi:10.1111/1759-7714.13488

23. Elkrief A, Richard C, Malo J, et al. Efficacy of immune checkpoint inhibitors in older

patients with non-small cell lung cancer: Real-world data from multicentric cohorts in Canada and France. *J Geriatr Oncol*. 2020;11(5):802-806.

24. Foster CC, Sher DJ, Rusthoven CG, et al. Overall survival according to immunotherapy

and radiation treatment for metastatic non-small-cell lung cancer: a National Cancer Database analysis. *Radiation Oncol*. 2019;14(1):18. Published 2019 Jan 28.

25. Huang L, Li L, Zhou Y, et al. Clinical Characteristics Correlate With Outcomes of

Immunotherapy in Advanced Non-Small Cell Lung Cancer. *J Cancer*. 2020;11(24):7137-7145. Published 2020 Oct 18. doi:10.7150/jca.49213

26. Kano H, Ichihara E, Harada D, et al. Utility of immune checkpoint inhibitors in non-small-cell lung cancer patients with poor performance status. *Cancer Sci*. 2020;111(10):3739-3746.

27, Lichtenstein MRL, Nipp RD, Muzikansky A, et al. Impact of Age on Outcomes with Immunotherapy in Patients with Non-Small Cell Lung Cancer. *J Thorac Oncol*. 2019;14(3):547-552.

28. Lin SY, Yang CY, Liao BC, et al. Tumor PD-L1 Expression and Clinical Outcomes in Advanced-stage Non-Small Cell Lung Cancer Patients Treated with Nivolumab or Pembrolizumab: Real-World Data in Taiwan. *J Cancer*. 2018;9(10):1813-1820. Published 2018 Apr 19.

29. Nazha B, Goyal S, Chen Z, et al. Efficacy and safety of immune checkpoint blockade in self-identified Black patients with advanced non-small cell lung cancer. *Cancer*. 2020;126(23):5040-5049.

30. Ng TL, Liu Y, Dimou A, et al. Predictive value of oncogenic driver subtype, programmed death-1 ligand (PD-L1) score, and smoking status on the efficacy of PD-1/PD-L1 inhibitors in patients with oncogene-driven non-small cell lung cancer. *Cancer*. 2019;125(7):1038-1049.

31, Prelaj A, Ferrara R, Rebuzzi SE, et al. EPSILoN: A Prognostic Score for Immunotherapy in Advanced Non-Small-Cell Lung Cancer: A Validation Cohort. *Cancers (Basel)*. 2019;11(12):1954. Published 2019 Dec 5.

32, Smit HJM, Aerts J, van den Heuvel M, et al. Effects of checkpoint inhibitors in advanced non-small cell lung cancer at population level from the National Immunotherapy Registry. *Lung Cancer*. 2020;140:107-112.

33. Song P, Yang D, Cui X, et al. NLCIPS: Non-Small Cell Lung Cancer Immunotherapy Prognosis Score. *Cancer Manag Res*. 2020;12:5975-5985. Published 2020 Jul 17.

34. Yang S, Zhang W, Chen Q, Guo Q. Clinical Investigation of the Efficacy and Safety of Anlotinib with Immunotherapy in Advanced Non-Small Cell Lung Cancer as Third-Line Therapy: A Retrospective Study. *Cancer Manag Res*. 2020;12:10333-10340. Published 2020 Oct 19. doi:10.2147/CMAR.S280096.
